# Factors associated with caretakers’ knowledge, attitude, and practices in the management of pneumonia for children aged five years and below in rural Uganda

**DOI:** 10.1186/s12913-023-09713-z

**Published:** 2023-06-27

**Authors:** Dan Kajungu, Betty Nabukeera, Michael Muhoozi, Donald Bruce Ndyomugyenyi, Mercy Consolate Akello, Collins Gyezaho, James Waako, Ronnie Kasirye

**Affiliations:** 1 Makerere University Centre for Health and Population Research (MUCHAP), Kampala, Uganda; 2 Department of Global Health, Stellenbosch University, Stellenbosch, 7602 South Africa; 3 Iganga General Hospital and Kamuli District, Kampala, Uganda

**Keywords:** Pneumonia, Knowledge, Attitude, Practice, Healthcare seeking, Uganda

## Abstract

**Background:**

Efforts aimed at reducing morbidity and mortality associated with pneumonia in children aged five years and below largely depend on caretakers. This study aimed to assess the factors associated with knowledge, attitudes, and practices of caretakers regarding pneumonia.

**Methods:**

This was a cross-sectional study carried out within Iganga and Mayuge health and demographic surveillance site (IMHDSS) cohort in Eastern Uganda. Caretakers of children under the age of five years were assessed for knowledge, attitudes, and practices using a set of indicators. The caretaker characteristics as determinants for knowledge, attitude, and practices in relation to pneumonia management were assessed for association. Logistic regression was used to assess the factors associated with caretaker knowledge, attitudes and practices.

**Results:**

A total of 649 caretakers of children five years and below of age were interviewed. Caretakers knew pneumonia as one of the childhood diseases, but were less knowledgeable about its transmission, signs and symptoms, risk factors and treatment. Overall, 28% had good knowledge, 36% had moderate knowledge and 35% had poor knowledge. The caretaker attitude was good for more than a half of the respondents (57%), while majority reported good practices (74.1%). Older age (OR = 1.63, 95% CI (1.05–2.51)), Tertiary education (OR = 4.92, 95% CI (2.5–9.65)), being married (OR = 1.82, 95% CI (1.05–3.15)) were associated with having good knowledge. Age above 35 years (aOR = 1.48, 95% CI (1.03–2.11)), and main source of livelihood were associated with good attitude and lastly being female (OR = 2.3, 95% CI (1.23–4.37)), being a Muslim (aOR = 0.5, 95% CI (0.35–0.75)), and being a farmer (OR = 0.5, 95% CI (0.33–0.85)) were associated with being a good caretaker practice.

**Conclusions:**

The caretakers of children five years and below, have relatively adequate knowledge about the signs and symptoms of pneumonia, risk factors and treatment measures. Higher education, being married, and being a salary earner were associated with better knowledge about pneumonia, while being female, being a Muslim, and being a peasant farmer were associated with good practice. Targeted interventions to equip caretakers with relevant and adequate skills and knowledge for lower-income and less educated caretakers, considering cultural and religious beliefs about childhood pneumonia identification and management are required.

## Introduction

Pneumonia is an acute infectious disease of the lungs caused by bacteria, viruses or fungi [1] and it can affect anyone although children 5 years and below are most susceptible [2]. The signs and symptoms associated with pneumonia include fever, cough, wheezing, chest pain, hypothermia, inability to feed or drink, fast breathing, difficulty in breathing, and in some cases convulsions [1, 3, 4].

Pneumonia remains responsible for more deaths among children under five years of age than any other infectious disease worldwide [3] with most of these deaths being registered in sub–Saharan Africa and Asia at 50% and 31% respectively of all worldwide under-five deaths [5]. Sadly, early and prompt care-seeking still remains a challenge in countries with high mortality rates [6]. Uganda has had a reduction in its under-five mortality rates from 56.1 deaths per 1000 live births in 2015 to 45.8 deaths per 1000 live births in 2019 [7], however despite these reductions, the under 5 mortality rate still remains significantly high with the leading causes of death being malaria, diarrhea and pneumonia, all of which can be easily prevented/treated using cost-effective measures [8, 9]. Children are often looked after by caretakers especially when they are ill. These are usually their mothers, aunties, grandparents, and siblings. The overwhelming majority of caretakers of children continue to have inadequate knowledge regarding pneumonia and other childhood illnesses, their signs and symptoms, as well as danger signs, the modes of transmission, risk factors, and prevention/treatment measures [3, 10]. This poses a big challenge to achieving the Sustainable Development Goal (SDG) 3, Target 3.2 which aims at ending preventable deaths of newborns and children under 5 years of age for all countries [11].

Although several studies have been done regarding pneumonia, there still exists an alarming gap in knowledge pertaining to the ability of caretakers to identify signs and symptoms, danger signs, modes of transmission, risk factors, and prevention/treatment measures for pneumonia as well as the associated attitudes of caretakers [3, 10, 12]. The World Health Organization and UNICEF have recommended strengthening family’s capacity to recognize danger signs and prompt care-seeking as one of the interventions for controlling pneumonia in children under five years of age [13].

In Uganda and Nigeria, delay in health-seeking has been attributed to sociodemographic factors, poor knowledge, poor attitudes, and poor practices towards the management of pneumonia as reported among caretakers as well as health workers [14, 15]. Inadequate knowledge about pneumonia like signs/symptoms, modes of transmission, risk factors, and prevention/treatment [14] are the most significant factors.

Recognition of signs and symptoms of pneumonia is the first and most important step towards reducing death among children five years and below. Caretakers play a significant role in recognizing these signs (in particular the danger signs) and symptoms, and in seeking appropriate care for the sick child [10]. Appropriate care includes consultation with providers who can correctly diagnose and treat pneumonia such as at health facilities and community-based health providers [10]. Although a number of studies have been done to assess Knowledge Attitude Practices (KAP) and risk factors of Pneumonia, there is a dearth of information on the factors driving those levels of knowledge, attitudes and practices among caretakers. Therefore, this study aimed to assess the factors associated with knowledge, attitude, and practices of caretakers regarding management of pneumonia in rural Uganda.

## Methods

### Study setting and target population

This study was nested within the Iganga Mayuge Health and Demographic Surveillance Site (IMHDSS) located in Eastern Uganda in 2019. The IMHDSS longitudinal population cohort was founded in 2005 and covers 65 villages in a clearly demarcated area within the Iganga and Mayuge districts. Currently, there are 101,302 residents from 17,429 households, with 60% living in rural and 40% in peri-urban areas. The IMHDSS catchment area includes 23 health facilities within the demographic surveillance area which include two general hospitals, 15 level II Health Centres (HCII) (11 public, 3 private-not-for-profit (PNFP) and 1 private-for-profit), 6 level III Health Centres (5 public and 1 PNFP), 1 level IV Health Centre (public) and over 20 private clinics. The cohort characteristics have been profiled elsewhere [16].

### Study design, sample size and sampling procedure

This was a cross-sectional study design.

### Sample size

A caretaker was defined as a person who was found to be in caring or regarded as a guardian of a child aged 5 years and below at the time of the study while basing on roles held. A minimum sample of caretakers of children aged five years and below was estimated using the Kish Leslie 1965 formulae [17].

### Sampling method

The study population was obtained using systematic sampling Systematic sampling was used to select households with the eligible caretaker of a child below five years of age. Using data from the IMHDSS population-based cohort, all villages were grouped into 25 enumeration areas. Within each enumeration area, 26 households were systematically selected. In larger villages (> 250 households), the study team randomly chose a direction from the center of the village, randomly selected the starting household from the first five houses in that direction, and then systematically selected every fifth house until they reached the edge of the village. In villages with less than 250 households, the total number was divided by the desired number of households to determine the sampling interval. Caretakers of children aged five years and below from those households were interviewed. The villages represented both urban and rural settings to provide better representation of the population.

### Data collection

#### Training

Data collectors were trained on study procedures by the team of investigators. The training covered data collection tools, field procedures and interview techniques.

#### Tool development

Pre-testing of the questionnaire and field procedures was done by the field researchers and supervisors who had attended the training and a debrief session. This was conducted following the pre-test to discuss challenges identified and changes made as a result of the pre-test. The study tool was translated into local language (Lusoga) and back translated into English to ensure that meaning is maintained but also the local implicit terms are not missed. The interviews were conducted with the caretakers in Lusoga. The original tool was designed thorough literature review and comparisons with different knowledge, attitude and practices surveys. Responses elicited information about childhood pneumonia, knowledge about signs and symptoms in children and actions taken by the caretaker in the management of childhood pneumonia.

#### Validation

A pilot study was conducted in another district with similar setting to improve the quality and validity of the data collected. All questionnaires were monitored for completeness and accuracy and validated by a research supervisor. Data were collected on knowledge about signs and symptoms, risk factors and treatment and management of childhood pneumonia, attitude of caretakers towards the health care system as well as their practices in relation to healthcare seeking.

#### Definition and measurement of outcome variables

##### Knowledge

Defined as theoretical or practical understanding of a subject. The caretaker was assessed on knowledge of childhood diseases and pneumonia by considering questions that were specific to the signs and symptoms, risk factors, mode of transmission, and treatment and/or management (see attached questionnaire).

##### Attitude

Defined as an established way of thinking or feeling about something. The caretaker’s attitude was assessed in relation to the health facility environment, health care system and processes for management of childhood pneumonia. The attitude indicators were assessed using a Likert scale of ‘Strongly disagree’, ‘Disagree’, ‘Neutral’, ‘Agree’ and ‘Strongly agree’.

##### Practice

The actual application of an idea, belief, or method, as opposed to theories relating to it. The practice section of the study tool consisted of statements relating to health care practices and the responses of each statement were assessed with a 3-point Likert scale ‘Never’, ‘Occasionally’ and ‘Always’.

#### Exposure variables

Socio-demographic variables including age, gender, educational level, marital status, religion, and socioeconomic/livelihood source were obtained through closed-ended questions.

### Data management and analysis

The data collection tool for the assessment of the factors associated with caretakers’ knowledge, attitude and practices towards management of Pneumonia among children aged 5 years and below was based on the questions adapted from published literature [18, 19]. The questionnaire was divided into 4 portions: the first portion deals with socio-demographic characteristics; the remaining three portions contain questions on the assessment of participants knowledge (12 questions), attitudes (17 questions) and practices (5 questions) of the caretakers. Knowledge, attitudes and practice scores of individuals were calculated to give the total knowledge, attitudes and practice score. The analysis of three modules was done on the basis of scalar-scoring method.

### Knowledge

The section assessing knowledge (12 questions (3 questions from each of the subsections: risk factors, transmission, symptoms and treatment)) were answered on a basis of mentioning it and an additional “I don’t know” option. A correct answer was assigned 1 point and an incorrect/unknown answer was assigned 0 point. The total knowledge score ranged from 0 to 12. Caretakers’ overall knowledge was categorized, using Bloom’s cut-off point, as good if the score was between 80 and 100% (9.6–12 points), moderate if the score was between 60 and 79% (7.2–9.6 points), and poor if the score was less than 60% (< 7.2 points).

### Attitude

Questions assessing attitude (17 questions) were assessed using a Likert scale of ‘Strongly disagree (1)’, ‘Disagree (2)’, ‘Neutral (3)’, ‘Agree (4)’ and ‘Strongly agree (5)’. The overall attitude score was categorized using the same Bloom’s cut-off point, as good if the score was between 80 and 100% (68-85points), moderate if the score was between 60 and 79% (51–67 points), and poor if the score was less than 60% (< 51 points).

### Practice

Practice of caregivers towards management of childhood Pneumonia was assessed by 5 questions using a 3-point Likert scale ‘Never (0)’, ‘Occasionally (1)’ and ‘Always (2)’. and the same Bloom’s cut off point was applied. Completed questionnaires were double entered using EPIDATA and imported into STATA 15 for cleaning and analysis.

Analysis consisted of graphical displays, descriptive statistics including frequency distribution, percentage, and the mean scores. A binary multiple logistic regression was used to assess the factors associated with caretaker knowledge, attitudes and practices. All the model point estimates with their 95% confidence interval were presented and interpreted.

### Ethical considerations

Ethical approval was obtained from the Mildmay Research Ethics Committee (MUREC) (REF: 0312–2019). Informed consent was obtained from all participants in the surveys and data anonymity was ensured. All respondents were informed about the content and the purpose of the survey, the expected time for participating, and that all information would be kept confidential. They were then asked to consent to participate in the study. Data are stored on a password-protected computer after identifiers had been removed. This study was conducted in accordance with international ethical standards (Declaration of Helsinki 1964).

## Results

A total of 649 respondents completed the survey questionnaire with a mean age of 30.3 (*SD* = 8.9) years. The majority of the participants were female (*n* = 600, 92.4%) and married (*n* = 537, 82.7%). More than a half of the respondents were Christians (57.4%) and the rest were Moslems (42.5%). Among the respondents, majority were peasant farmers (57.3%), 23.7% were not working, while the rest (18.9%) were salary/wage earners as shown in Table 1.

**Table 1:** Sociodemographic characteristics of the respondents

Characteristics	Frequency (*n* = 649)	Percentage (%)
**Sex of Respondents**		
Male	49	7.55
Female	600	92.45
**Age Groups**		
15–34	472	72.73
35 and above	177	27.27
**Education Level**		
None	158	24.35
Primary	256	39.45
Secondary	128	19.72
Tertiary/University	107	16.49
**Religion**		
Christians	373	57.47
Muslims	276	42.53
**Marital Status**		
Unmarried	112	17.26
Married	537	82.74
**Main Source of Livelihood**		
Not working	154	23.73
Salary/Wage Earner	123	18.95
Peasant Farmer	372	57.32
**Source of health care for common childhood illnesses**		
Health facility/hospital	595	91.7
Pharmacy/drug shop	6	0.9
Private clinic	48	7.4

### The Knowledge, attitude, and practice in management of pneumonia

**Fig. 1:**
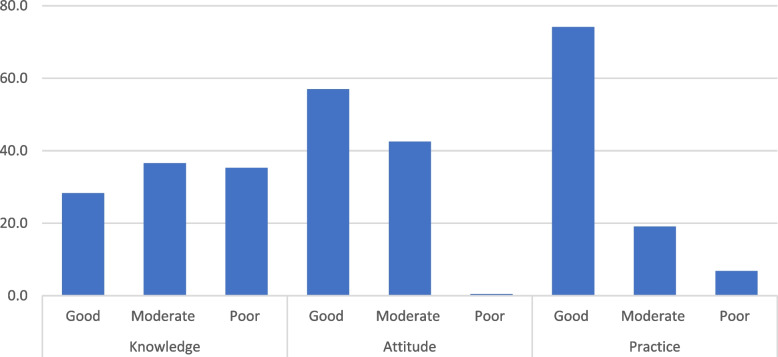
Proportion of Caretaker as ranked by their knowledge, attitude, and practice in management of pneumonia

The management of pneumonia by caretakers in terms of knowledge attitude and practice as shown in Fig. 1. Twenty eight percent of the caretakers reported good knowledge in management of pneumonia, 36% reported moderate while 35% reported poor. Attitude was reported as good by 57% of caretakers while 74% of the caretakers reported good practice (Fig. 1).

### Caretaker knowledge assessment

#### Awareness of all childhood illnesses

All respondents were aware or knowledgeable of at least one childhood illness and malaria was the most commonly known childhood illness. Malaria was mentioned by 89% (95%CI:86–91) of all caretakers, followed by diarrhea 46% (95%CI:42–50) while Pneumonia was mentioned by 21% (95%CI:19–24) of the respondents.

### Caretaker knowledge of pneumonia risk factors

**Fig. 2:**
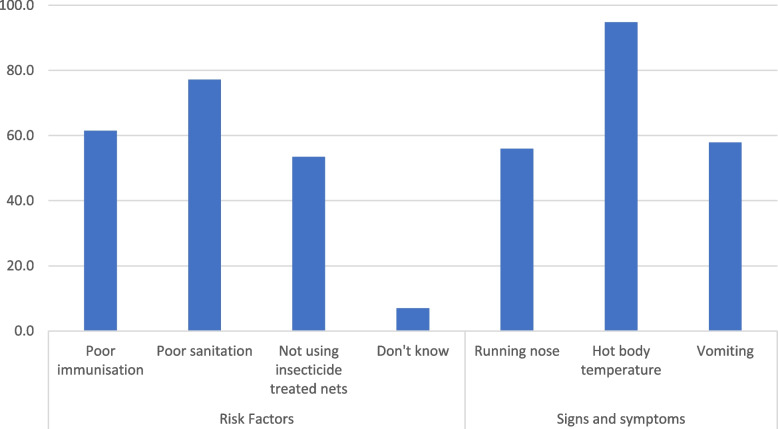
Caretaker knowledge of pneumonia risk factors as well as signs and symptoms

77% of caretakers mentioned poor sanitation as a risk factor for Pneumonia, 61% mentioned incomplete or no immunisation dose and 53% mentioned not sleeping under an insecticide treated mosquito net as risk factors for Pneumonia. 7%% of the caretakers did not know any risk factor (Fig. 2).

### Knowledge of signs and symptoms of pneumonia

The most commonly mentioned symptom was hot (elevated) body temperature, (mentioned by 95% of the caretakers) as shown in Fig. 2. This was followed by vomiting (58%), and running nose (56%).

### Knowledge of transmission routes

65% of the respondents mentioned airborne spread, 54% mentioned direct contact, while 49% mentioned indirect contact and there were 5% of the respondents who didn’t know any way in which pneumonia is transmitted.

### Knowledge of treatment for pneumonia

The most reported treatment for management of pneumonia was coartem (mentioned by 92% of the caregivers), followed by ORS solution (60%) and Amoxicilin (40%).

### Caretakers’ attitude towards management of pneumonia at the health Centre

**Fig. 3:**
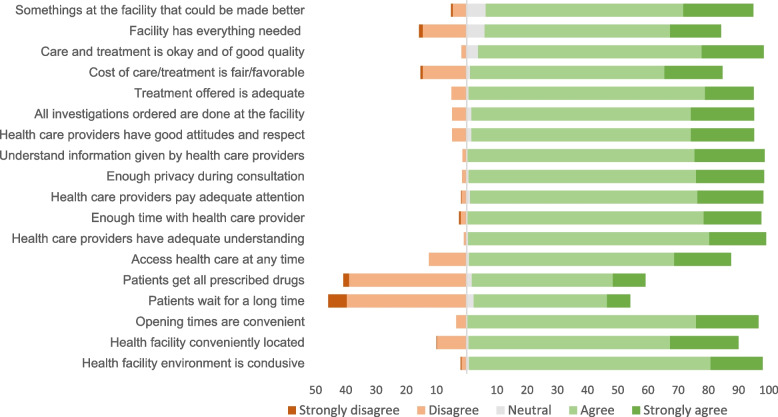
Attitude indicators scale as reported by the caretakers

More than half of the respondents (57%) had good attitude towards the management of Pneumonia. 42% had moderate attitude and only 3 caretakers had poor attitude (Fig. 1). The mean attitude score was 68.3 (SD: 6.4). The caretaker attitude assessment is highlighted in Fig. 3. Almost four out of every ten caretakers disagreed or strongly disagreed with the statement that patients get all prescribed drugs from the facility (41%) while one in every ten disagreed with the statement that a child can access health care at any time (12%). Close to 19% of the care takers were either not sure or disagreed with the statement that the facility has everything needed for treatment while 14% did not think that the cost of treatment was fair.

### Caretakers’ practice towards the management of pneumonia

**Fig. 4:**
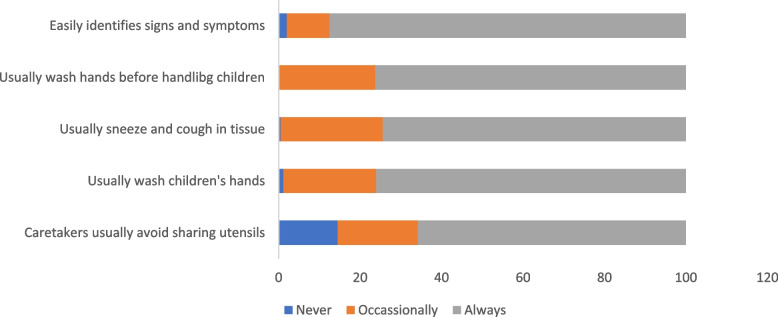
The practice indicators scale as reported by caretakers

Majority of the caretakers reported good practice (74.1%), 19.1% had moderate practices and only 6.8% had poor practice towards the management of pneumonia. The assessment of practice is highlighted by the indicators shown in Fig. 4. Around 33% of caretakers indicated that it is not easy to avoid sharing kitchen utensils. Close to 25% of the caretakers reported some level of difficulty in sneezing and coughing in tissue all the time, washing a child’s hand before eating, and washing hands before handling a child.

Caretakers that were aged 35 years and above had better knowledge about pneumonia (aOR: 1.63(95% CI:1.05 – 2.51)), and this was similar for married participants compared to unmarried (aOR: 1.82(95%CI:1.05 – 3.15)). Participants with higher education levels especially those with post-secondary (Tertiary) had more knowledge about pneumonia in this population compared to those who indicated none as their education level. The participants whose source of livelihood was salary/wage earners were more likely to know about pneumonia compared to those who were not working as shown in Table 2. For the determinants of attitude, it was married caretakers who had better attitudes compared to unmarried ones (aOR: 1.78 (95%CI:1.16 – 2.73)). This was also true for caretakers whose source of livelihood was a farmer (aOR: 2.50(95%CI: 1.38 – 4.52)) as well as salary/wage earners (aOR: 1.75(95%CI: 1.13 – 2.72)). Older caretakers showed a 32% probability of having a better attitude, although this was not statistically significant (aOR:1.32(95%CI:0.89 – 1.97)) as shown in Table 2. Exploring the determinants of caretaker practices in the management of pneumonia showed that a female caretaker was more likely to demonstrate appropriate practices compared to male caretakers (aOR: 2.77 (95%CI:1.23 – 4.37)). On the other hand, older caretakers were less likely to have proper practices compared to young caretakers, but this difference was not statistically significant as shown in Table 2. Education was another determinant of good practices where caretakers with Tertiary level of education had higher odds compared to those with no education. Being a Muslim caretaker was associated with having lower odds of being knowledgeable, having a good attitude, or applying appropriate practices in the management of pneumonia in this population compared to Christian caretakers but this was not statistically significant as shown in Table 2.

**Table 2:** Multiple logistic regression analysis for the determinants of knowledge, attitude, and practice

**Variable**	**Knowledge**a**ble**		**Attitude**		**Practice**	
	**OR [95%CI]**	**aOR [95%CI]**	**OR [95% CI]**	**aOR [95%CI]**	**OR [95%CI]**	**aOR [95%CI]**
**Sex**						
Male		Ref		Ref		Ref
Female	1.39[0.69–2.78]	1.55[0.73–3.31]	0.68[0.37–1.26]	0.81[0.43–1.55]	2.77[1.54–5.01]	2.32[1.23–4.37]
**Age (in years)**						
15–34		Ref		Ref		Ref
35 and above	1.56[1.08–2.27]	1.63[1.05–2.51]	1.48[1.03–2.11]	1.32[0.89–1.97]	0.75[0.51–1.11]	0.89[0.57–1.39]
**Marital status**						
Unmarried		Ref		Ref		Ref
Married	2.0[1.19–3.36]	1.82[1.05–3.15]	0.81[0.59–1.10]	1.78[1.16–2.73]	1.38[0.88–2.15]	1.40[0.87–2.25]
**Education level**						
None		Ref		Ref		Ref
Primary	0.90[0.56–1.46]	1.05[0.62–1.76]	0.81[0.54–1.21]	1.00[0.64–1.54]	1.39[0.91–2.13]	1.01[0.68–1.77]
Secondary	0.99[0.57–1.73]	1.05[0.56–1.98]	0.74[0.46–1.18]	0.98[0.57–1.69]	1.54[0.92–2.57]	0.88[0.48–1.62]
Tertially	5.04[2.95–8.62]	4.91[2.50–9.65]	1.84(1.09–3.12)		5.81[2.73–12.40]	3.20[1.32–7.69]
**Religion**						
Christians		Ref		Ref		Ref
Muslims	0.60[0.42–0.86]	0.75[0.51–1.10]	0.81[0.59–1.10]	0.95[0.68–1.32]	0.48[0.33–0.68]	0.53[0.36–0.77]
**Main livelihood source**						
Not working		Ref		Ref		Ref
Salary/Wage Earner	3.15[1.87–5.29]	1.17[0.62–2.22]	3.68[2.21–6.12]	2.50[1.38–4.52]	1.62[0.84–3.13]	1.83[0.39–1.76]
Farmer	1.14[0.73–1.79]	1.10[0.65–1.85]	1.97[1.34–2.8]	1.75[1.13–2.72]	0.49[0.31–0.78]	0.50[0.29–0.85]

Note: *OR* Odds Ratio, *aOR* Adjusted Odds Ratio, *CI* Confidence Interval, *Ref* Reference

## Discussion

This study looked at factors associated with knowledge, attitude, and practices of caretakers for children five years and belowin rural Uganda. Knowledgeable caretakers with good attitude and right practices in the management of pneumonia greatly contribute to mitigating the disease burden, reduce morbidities and mortalities as well as the cost and burden on the healthcare system in low- and middle-income countries.

### Caretaker knowledge

A third of the caretakers knew pneumonia as a common childhood illness. This is important since perceptions about how common a disease is; may have an influence on interpretation of severity, cause and actions taken by the caretakers in seeking health care [20]. The poor caretaker knowledge of the mode of transmission, risk factors, signs and symptoms and treatment of pneumonia, was similar to findings from studies done in Bangladesh and Mumbai where the majority could not recognize whether their child had Pneumonia or not [21–24].

Among the Integrated Management of Childhood Illnesses (IMCI) standardized danger signs, incessant vomiting was the most cited followed by convulsions. However, no caretaker mentioned all the four standardized danger signs. Chest wall in-drawing/ fast breathing and hot body temperature were identified as signs and symptoms of pneumonia and was more than what was found in Mirpurhas, Pakistan where fast breathing & chest in-drawing were reported as symptoms for pneumonia by 59.4% of mothers [25]. Other studies also reported that participants correctly identified fast/difficult breathing as being suggestive of pneumonia [26–28] and none of the caretakers mentioned all the danger signs. This is likely due to the low literacy levels among the caretakers in this study area where some of the caretakers could not clearly identify a single sign and symptom. This is an indication that more effort is needed in sensitizing caretakers to recognise the signs and symptoms of pneumonia and other childhood illnesses and the importance of seeking early medical care.

Caretakers with higher education levels had better knowledge about Pneumonia and other childhood illnesses. This could be explained by the caretakers’ exposure to different sources of information which is consistent with findings reported by Duke et al. [26], who reported that mothers with higher educational levels had better knowledge about Pneumonia and other childhood illnesses.

Older age was significantly associated with being knowledgeable about pneumonia. This is concurrent with study conducted in Kenya [18] and other studies conducted elsewhere [31, 28]. This could be because of increased exposure to information about pneumonia over time, higher levels of education, or greater personal or professional experiences with the disease. Being married was also associated with being good of knowledge about pneumonia concurrent with study conducted in Denmark that reported that married individuals have a decreased risk of being hospitalized with pneumonia compared with never-married, divorced, and widowed patients [29]. However, a study conducted in India did not find any significant association between being married and knowledge of pneumonia signs and symptoms [19]. This could be because the study was conducted in contextually different environment and assessed 8 factors such as simple signs and symptoms, assessment, prevention, causes and factors. Interventions should take into account the potential role of social support and social networks in promoting knowledge of pneumonia among caretakers of children [30].

Additionally, being a salary earner was associated with good being knowledgeable concurrent a prospective population-based study conducted in Brazil which found an association between higher income and lower risk of developing pneumonia [31]. Given that 42% of households in Uganda experience catastrophic health expenditures based on their monthly income for an episode of pneumonia in children under five years old [32] There is need to target education and awareness efforts towards populations who are less likely to be employed or earning a salary.

The household-specific factors of poor sanitation, overcrowding and use of biofuels were identified as risk factors of acquiring Pneumonia. Other studies reported poor hygiene practices as a risk factor for developing pneumonia [12], while household crowding was seen to double the likelihood of developing pneumonia [33] and, the use of solid fuel for cooking which causes indoor air pollution was also associated with increased risk of pneumonia [34].

On the other hand, a child-specific risk factor was poor immunisation status while caretaker-specific risk factors included low parent’s education level and smoking status of a parent. There are other studies which found poor immunisation status to be associated with an increased risk of developing pneumonia [33, 35], and lack of education for the mother or father also was associated with high risk of pneumonia [34, 36].

All the caretakers were able to state at least one treatment measure. Use of Antibiotics(like Amoxicillin, Cotrimoxazole and Gentamycin) was identified by caretakers for the treatment of childhood illnesses which is similar to studies where caretakers mentioned antibiotics (like Cotrimoxazole and Amoxicillin) as the commonly used treatment for pneumonia [37, 38]. The majority of caretakers mentioned Paracetamol, ORS and Vitamin A supplement which is similar to findings by other researchers where caretakers mentioned Paracetamol and supplements like ORS, and vitamin C as treatment for childhood illnesses in children five years and below [4, 39]. This was however different from Minz et al. who found out that Vitamin A supplement was perceived as unimportant in prevention and treatment of childhood illnesses [36].

### Attitude towards treatment and healthcare seeking

Majority of the respondents in this study had good attitude similar findings were reported by Gundluru et al. [39]. This was however contrary to some studies which reported unfavorable caretakers’ attitude [14, 40]. Patients get all prescribed drugs from the facility as expected since most of the care givers sought care/treatment at public health facilities that offer free services. In other situations caretakers were frequently advised to buy drugs, needles or infusion fluids outside the health facility [14]. There was a significant relationship between marital status and attitude towards care seeking which is consistent with Alene et al. who reported that married mothers had an increased likelihood of seeking health care, unlike those mothers who were not living with their husbands [41]. This finding is probably due to the fact that caring for a child by oneself can be socially and financially burdensome compared to sharing responsibilities. Similar to good knowledge, the study also found an association between being a salary earner and having good attitude of caretakers regarding management of pneumonia as with other studies [19, 32] Most of the respondents in our study agreed that the cost of care/treatment is fair and favorable. This is expected since majority sought care at government health facilities in which care/treatment is offered free of charge. This was however different for Hildenwall et al. [14] as well as Bakare et al. [15] who found an existing financial constraint to care-seeking for sick children. Bakare et al. [15] further reported that this financial barrier was related to the purchase of drugs after a diagnosis had been made.

In the case of health care seeking, almost half of the respondents agreed that patients waited for a long time to receive health care which agrees with Hildenwall et al. [14] findings that the patients waiting time before being attended to was generally longer at hospitals. c More respondents agreed that healthcare providers who treated sick children had good attitude and they were respectful which was contrary to the findings by Hildenwall et al. who argued that some caretakers stated indifference among health care providers as well as rude reception [14]. A similar conclusion was reached at in Nigeria by Bakare et al. who reported that although caregivers in Jigawa and Lagos accepted and valued the care provided to them at primary health care facilities, there were accounts of dissatisfaction with the health workers’ attitude [15].

### Treatment and healthcare seeking practices

Being Muslim, female and a peasant farmer were significantly associated with good practices of caretakers regarding management of pneumonia. This consistent with studied conducted in contextually setting that found religion [42, 43] gender [44, 45] and occupation [46, 47] as important determinants for good practices for caretakers regarding management of pneumonia. Practice by caretakers was good because they always brought children for immunization when it was due which differed from other studies which found out that practice among caregivers was unsatisfactory [37]. Others have argued that, even with good immunization uptake, there were cases of missed doses of vaccines where children aged five years and below were either partially immunized or had never been immunized [35, 39]. Most caretakers always provided treatment to sick children as directed by health care providers a few reported that they occasionally or never provided treatment to sick children as directed. Similar findings were reported by Athumani et al. in a study conducted in Dar es Salaam [48], where most of the mothers ceased giving medication to children before dose completion.

The caretakers always promptly took their children to health facilities whenever they got ill. This is an indicator that women in this community are able to make good judgement and take appropriate actions towards healthcare-seeking for children. This was contrary to other studies that reported a delay in care seeking for ill children and attributing it to absence of the father who is considered the ultimate decision maker in the home as well as the influence of neighbors, friends and relatives who give suggestions as to what type of illness the child is suffering from [14, 15]. Even when the woman was able to identify the problem early enough, she would still be constrained by limited power and ownership of financial resources in the household which means that she still needs to consult with the husband before any action can be taken [15]. In other instances, the healthcare was only sought when the disease was perceived to be severe but most caretakers reported difficulty finding transport to go to the hospital [14]. Distance from the health facility, lack of belief in allopathy, financial constraints, poor recognition of severity of illness or danger signs, lack of family support and the primary decision maker regarding care seeking during an illness in the family being the father [39] were some of the barriers to prompt seeking of health care.

Adherence to hospitalization schedules for sick children as guided by health care providers was prominent, with a few cases occasionally or never adhering to given schedules. In another study, some caretakers reached a health care provider and still withdrew the child from initiating care due to a lack of money for continued care and the anticipation of high cost of transporting a dead body [14].

There were limitations to this study including relying on self-reported answers, which may be subject to recall and reporting bias. There was also the possibility of unidentified predictors or confounders regarding attitude and perceptions of caretakers about illness and health seeking behavior. Further research is needed to address these limitations.

## Conclusion

The caretakers of children five years and below, have relatively adequate knowledge about the signs and symptoms of pneumonia based on the IMCI guidelines or the risk factors and treatment measures. This study found that higher education, being married, and being a salary earner were associated with better knowledge about pneumonia, while being female, being a Muslim, and being a peasant farmer were associated with good practice. Targeted interventions to equip caretakers with relevant and adequate skills and knowledge for lower-income and less educated caretakers, considering cultural and religious beliefs about childhood pneumonia identification and management are required.

### Recommendations

Comprehensive interventions for increasing symptom recognition and improving health-seeking behavior are needed. Improving health literacy will have a significant impact on the reduction of childhood mortality. Women empowerment and counselling of family members is crucial to reduce childhood mortality.

Engagement of fathers for their involvement in childcare including education of danger signs especially when teaching mothers during antenatal visit sessions and during immunization visits.

## Data Availability

Data are stored at Makerere University Centre for Health and Population Research (MUCHAP). The datasets available from the corresponding author on reasonable request.

## References

[1] Unicef (2016). One is too many: ending child deaths from pneumonia and diarrhoea.

[2] Karo SJ, Lizarondo L, Stern C (2019). Caregivers’ and healthcare workers’ experiences in the management of childhood pneumonia in low-and lower middle-income countries: a qualitative systematic review protocol. JBI Evid Synth.

[3] Noordam AC, Sharkey AB, Hinssen P, Dinant G, Cals JWL (2017). Association between caregivers’ knowledge and care seeking behaviour for children with symptoms of pneumonia in six sub-Saharan African Countries. BMC Health Serv Res.

[4] Ndu IK, Ekwochi U, Osuorah CD, Onah KS, Obuoha E, Odetunde OI (2015). Danger signs of childhood pneumonia: caregiver awareness and care seeking behavior in a developing country. Int J Pediatr.

[5] You D, Hug L, Ejdemyr S, Idele P, Hogan D, Mathers C (2015). Global, regional, and national levels and trends in under-5 mortality between 1990 and 2015, with scenario-based projections to 2030: a systematic analysis by the UN inter-agency group for child mortality estimation. The Lancet.

[6] Herbert HK, Lee AC, Chandran A, Rudan I, Baqui AH (2012). Care seeking for neonatal illness in low-and middle-income countries: a systematic review. PLoS Med.

[7] Unicef (2016). data: monitoring the situation of children and women.

[8] World Health Organization. Children: improving survival and well-being. September 8, 2020. A WHO Fact sheet. Available on: https://www.who.int/news-room/factsheets/detail/children-reducing-mortality.

[9] Jamison DT, Breman JG, Measham AR, Alleyne G, Claeson M, Evans DB (2006). Cost-effective strategies for the excess burden of disease in developing countries. Priorities in Health.

[10] Tuhebwe D, Tumushabe E, Leontsini E, Wanyenze RK (2014). Pneumonia among children under five in Uganda: symptom recognition and actions taken by caretakers. Afr Health Sci.

[11] Morton S, Pencheon D, Squires N (2017). Sustainable Development Goals (SDGs), and their implementationA national global framework for health, development and equity needs a systems approach at every level. Br Med Bull.

[12] Abbey M, Chinbuah MA, Gyapong M, Bartholomew LK, van den Borne B (2016). Community perceptions and practices of treatment seeking for childhood pneumonia: a mixed methods study in a rural district Ghana. BMC Public Health.

[13] World Health Organization (2009). Global action plan for prevention and control of pneumonia (GAPP).

[14] Hildenwall H, Tomson G, Kaija J, Pariyo G, Peterson S (2008). “ I never had the money for blood testing”–Caretakers’ experiences of care-seeking for fatal childhood fevers in rural Uganda–a mixed methods study. BMC Int Health Hum Rights.

[15] Bakare AA, Graham H, Agwai IC, Shittu F, King C, Colbourn T (2020). Community and caregivers’ perceptions of pneumonia and care-seeking experiences in Nigeria: a qualitative study. Pediatr Pulmonol.

[16] Kajungu D, Hirose A, Rutebemberwa E, Pariyo GW, Peterson S, Guwatudde D (2020). Cohort profile: the Iganga-Mayuge health and demographic surveillance site, Uganda (IMHDSS, Uganda). Int J Epidemiol.

[17] World Health Organization (2016). WHO recommendations on antenatal care for a positive pregnancy experience.

[18] Amuka DO, Onguru D, Ayodo G. Knowledge, Perceptions and Practices of Caregivers on Pneumonia among Children aged below 5 Years in Migori County Referral Hospital, Kenya. 2020. Int J Health Sci Res. 2020;10(11):40–57.

[19] Pradhan SM, Rao AP, Pattanshetty SM, Nilima A (2016). Knowledge and perception regarding childhood pneumonia among mothers of under-five children in rural areas of Udupi Taluk, Karnataka: A cross-sectional study Indian. J Health Sci Biomed Res Kleu.

[20] Källander K, Tomson G, Nsabagasani X, Sabiiti JN, Pariyo G, Peterson S (2006). Can community health workers and caretakers recognise pneumonia in children? Experiences from western Uganda. Trans R Soc Trop Med Hyg.

[21] Ferdous F, Ahmed S, Das SK, Malek MA, Das J, Faruque ASG (2014). Mothers’ perception and healthcare seeking behavior of pneumonia children in rural Bangladesh. Int Schol Res Not.

[22] Black RE, Cousens S, Johnson HL, Lawn JE, Rudan I, Bassani DG (2010). Global, regional, and national causes of child mortality in 2008: a systematic analysis. The lancet.

[23] Chisti MJ, Ahmed T, Faruque ASG, Salam MA (2010). Clinical and laboratory features of radiologic pneumonia in severely malnourished infants attending an urban diarrhea treatment center in Bangladesh. Pediatr Infect Dis J.

[24] Kambli S (2014). Knowledge of bronchopneumonia among caretakers of infants. Int J Sci Res.

[25] Memon KN, Shaikh K, Pandhiani BS, Usman G (2013). How do mothers recognize & treat pneumonia in their children at home? A study in union council Jhudo, District Mirpurkhas. JLUMHS.

[26] Ekure E, Esezobor C, Balogun M, Mukhtar-Yola M, Ojo O, Emodi I (2013). Mothers and childhood pneumonia: what should the focus of public campaigns be?. Nigerian J Paediatr.

[27] Kapoor S, Reddaiah V, Murthy G (1990). Knowledge, attitude and practices regarding acute respiratory infections. Ind J Pediatr.

[28] Bham SQ, Saeed F, Shah MA (2016). Knowledge, Attitude and Practice of mothers on acute respiratory infection in children under five years. Pakistan J Med Sci.

[29] Mor A, Ulrichsen SP, Svensson E, Berencsi K, Thomsen RW (2013). Does marriage protect against hospitalization with pneumonia? A population-based case-control study. Clin Epidemiol.

[30] Cillóniz C, Greenslade L, Dominedò C, Garcia-Vidal C (2020). Promoting the use of social networks in pneumonia. Pneumonia.

[31] Thörn LK, Minamisava R, Nouer SS, Ribeiro LH, Andrade AL (2011). Pneumonia and poverty: a prospective population-based study among children in Brazil. BMC Infect Dis.

[32] Ekirapa-Kiracho E, De Broucker G, Ssebagereka A, Mutebi A, Apolot RR, Patenaude B (2021). The economic burden of pneumonia in children under five in Uganda. Vaccine X.

[33] da Fonseca Lima EJ, Mello MJG, Lopes MIL, Serra GHC, Lima DEP, Correia JB (2016). Risk factors for community-acquired pneumonia in children under five years of age in the post-pneumococcal conjugate vaccine era in Brazil: a case control study. BMC Pediatr.

[34] Mahalanabis D, Gupta S, Paul D, Gupta A, Lahiri M, Khaled M (2002). Risk factors for pneumonia in infants and young children and the role of solid fuel for cooking: a case-control study. Epidemiol Infect.

[35] Aguti B, Kalema G, Lutwama DM, Mawejje ML, Mupeyi E, Okanya D, et al. Knowledge and perception of caregivers about Risk factors and Manifestations of Pneumonia among under five children in Butaleja district, Eastern Uganda. Microbiol Res J Int. 2018;25(3)1–11.

[36] Minz A, Agarwal M, Singh JV, Singh VK, Sahu R (2019). Caregiver’s knowledge about childhood pneumonia: a study from rural areas and urban slums of Lucknow. Natl J Comm Med.

[37] Källander K, Hildenwall H, Waiswa P, Galiwango E, Peterson S, Pariyo G (2008). Delayed care seeking for fatal pneumonia in children aged under five years in Uganda: a case-series study. Bull World Health Organ.

[38] Ngocho JS, Horumpende PG, de Jonge MI, Mmbaga BT (2020). Inappropriate treatment of community-acquired pneumonia among children under five years of age in Tanzania. Int J Infect Dis.

[39] Gundluru M, Gopal H (2019). Knowledge, attitude and practice among mothers regarding common childhood illness. Int J Contemp Pediatr.

[40] Mekonnen GK, Mengistie B, Sahilu G, Mulat W, Kloos H (2018). Caregivers’ knowledge and attitudes about childhood diarrhea among refugee and host communities in Gambella Region, Ethiopia. J Health Popul Nutr.

[41] Alene M, Yismaw L, Berelie Y, Kassie B (2019). Health care utilization for common childhood illnesses in rural parts of Ethiopia: evidence from the 2016 Ethiopian demographic and health survey. BMC Public Health.

[42] Mebratie AD, Van de Poel E, Yilma Z, Abebaw D, Alemu G, Bedi AS (2014). Healthcare-seeking behaviour in rural Ethiopia: evidence from clinical vignettes. BMJ Open.

[43] Noordam AC, Carvajal-Velez L, Sharkey AB, Young M, Cals JW (2015). Care seeking behaviour for children with suspected pneumonia in countries in sub-Saharan Africa with high pneumonia mortality. PLoS ONE.

[44] Colvin CJ, Smith HJ, Swartz A, Ahs JW, de Heer J, Opiyo N (2013). Understanding careseeking for child illness in sub-Saharan Africa: a systematic review and conceptual framework based on qualitative research of household recognition and response to child diarrhoea, pneumonia and malaria. Soc Sci Med.

[45] Pandey A, Sengupta PG, Mondal SK, Gupta DN, Manna B, Ghosh S (2002). Gender differences in healthcare-seeking during common illnesses in a rural community of West Bengal, India. J Health Popul Nutr.

[46] Opuba EN, Owenga JA, Onyango PO (2021). Home-based care practices and experiences influencing health-seeking behaviour among caregivers of children diagnosed with pneumonia in Endebess Sub-County Kenya. J Global Health Rep.

[47] Yimenu DK, Kasahun AE, Chane M, Getachew Y, Manaye B, Kifle ZD (2022). Assessment of knowledge, attitude, and practice of child caregivers towards oral rehydration salt and zinc for the treatment of diarrhea in under 5 children in Gondar town. Clin Epidemiol Global Health.

[48] Athumani J (2008). Knowledge, Attitudes and Practices of mothers on symptoms and signs of integrated management of Childhood Illnesses (IMCI) strategy at Buguruni Reproductive and Child Health clinics in Dar es Salaam. Dar Es Salaam Med Stud J.

